# Effect of plasma homocysteine on cardiometabolic multimorbidity among Chinese adults: a population-based and real-world evidence study

**DOI:** 10.3389/fnut.2024.1522212

**Published:** 2024-12-20

**Authors:** Ling Li, Jia Wang, Jing Li, Minqi Li, Jie Wang, Tianyao Long, Yangyi Zhengliu, Xuan Tan, Yiwei Peng, Xiuqin Hong

**Affiliations:** ^1^Clinical Epidemiology Research Office, Hunan Provincial People's Hospital, The First Affiliated Hospital of Hunan Normal University, Changsha, China; ^2^Cerebral Vascular Disease Rehabilitation Clinical Research Center, Hunan Provincial People's Hospital, The First Affiliated Hospital of Hunan Normal University, Changsha, China; ^3^Department of Scientific Research, Hunan Provincial People's Hospital, The First Affiliated Hospital of Hunan Normal University, Changsha, China; ^4^Key Laboratory of Molecular Epidemiology, Hunan Normal University, Changsha, China

**Keywords:** homocysteine, cardiometabolic multimorbidity (CMM), Chinese adults, population-based study, real-world evidence

## Abstract

**Aims:**

To explore the effect of plasma homocysteine (Hcy) on cardiometabolic multimorbidity (CMM) among Chinses adults.

**Methods:**

This study combined a community-based cross-sectional study with a 1:1 matched case–control study using propensity score method among adults aged over 30 years in six districts randomly selected from Hunan Province, China. We recruited 5,258 people, of whom 4,012 met the study criteria were enrolled. CMM was defined as the coexistence of two or more cardiometabolic diseases, including diabetes, hypertension, coronary heart disease and stroke. The plasma Hcy and other laboratory data was measured by chemical automatic detector. Lifestyles and personal characteristics were collected by a questionnaire. Multivariate models were used to explore the associations. We calculated the attributable risk proportion (ARP) for the association of Hcy with CMM. The dose–response relationship was evaluated using restricted cubic splines method.

**Results:**

Of the 4,012 adults, 436 had CMM, with a population prevalence of 10.9%. In the propensity-score-matched case–control study, 828 (414 cases and 414 controls) were included, and those with high plasma Hcy level (>16.2 μmol/L) had a higher risk of CMM than those with lowest level (<10.4 μmol/L) (adjusted OR = 2.83, 95% CI: 1.84–4.36, *p* < 0.001), with a multivariate ARP of high level of exposure was 64.66% (95% CI: 46.24–77.06%). The largest effect combination of CMM was the coexisting of diabetes, hypertension and coronary heart disease (adjusted OR = 2.26, 95%CI: 1.43–3.57, *p* < 0.001). An inverse association and dose–response relationship were observed between CMM and plasma Hcy levels. Notably, we recognized a significant mediation effect by C-reactive protein, total cholesterol, triglyceride and waist circumference, and they mediated approximately 8 ~ 23% of the effect of Hcy on risk of CMM.

**Conclusion:**

Our findings add new evidence to this field that of high level of plasma Hcy was consistently associated with higher risk of CMM among Chinses adults, with the largest effect combination of being coexisting diabetes, hypertension and coronary heart disease. These findings have implications for cardiologists that CMM can be attributable to high level of plasma Hcy, and for decision makers that Hcy has become a public threat that persistently affects cardiovascular health in humans.

## Introduction

Globally, cardiometabolic multimorbidity (CMM) referring to the coexistence of two or more cardiometabolic diseases (CMDs), including diabetes, hypertension, coronary heart disease (CHD) and stroke, is closely related to decreased quality of life and increased economic burden of disease (1–3).

Currently, scientists became interested in new risk factors for CMDs. Here, plasma homocysteine (Hcy), a sulfur-containing amino acid, an intermediate product of methionine metabolism, has been identified as a newly discovered potential risk factor for a variety of diseases including diabetes, hypertension, neurodegenerative diseases, osteoporosis, and cancer (4). Its mechanisms refer to inflammatory response and oxidative stress, vascular endothelial cells damage, prothrombotic, pro-smooth muscle cell proliferation, methylation, and lipid metabolism disorder *in vivo* (5). Numerous epidemiological studies have established the associations between an increase in plasma Hcy levels and CMDs. For instance, an 11-year follow-up study from NHANES found that Hcy was a promising biomarker in risk stratification among diabetic patients (4). Additionally, it has been shown that 75% of hypertension cases also have hyper-Hcy (HHcy), called H-type hypertension, which was first introduced by Chinese researcher in 2008 (6, 7). The risk of CVDs in hypertension patients with HHcy was approximately 5 and 12 times that of single-hypertension and healthy people, respectively (8), and HHcy was independently associated with atherosclerotic plaques and stroke (9). Although the associations between Hcy and single CMDs are well-established, a limited number of studies examined the associations between Hcy and CMM. So far, only one study has reported an association between Hcy and CMM, while no positive result has been found (10).

Studies have confirmed that the generation of Hcy is completely dependent on the methionine cycle metabolism pathway, and human methionine is completely obtained from food, so the influence of diet on HCY cannot be ignored (11). The association between Hcy and CMM will be differed by confounders level (such as diet and lifestyles, etc.) in different directions and magnitudes. Thereby, a case–control study based on propensity-score-matched method was conducted in this study, using a semiparametric approach to increase the likelihood of a reasonable match between the case and the control group, and dealing with multiple confounders or stratification, greatly improving the reliability of the results (12). Additionally, the prevalence of HHcy is much higher among adults in China than in other countries (13–15). And most of epidemiological studies only focused on hospital-based populations, with few based on general community populations, which may limit interpretation of these data. Those were mainly concentrated in North China and rare in South China (14). Thus, we conducted a community-based, propensity-score-matched, and case–control study to obtain the real-word evidence of the association between Hcy and CMM by investigating the multi-aspects influencing factors of CMM, to determine the common biomarkers of CMM, and to provide a scientific basis for the preventive and therapeutic strategies of CMM in Chinese adults.

## Methods

### Study design and sample

**Figure 1:**
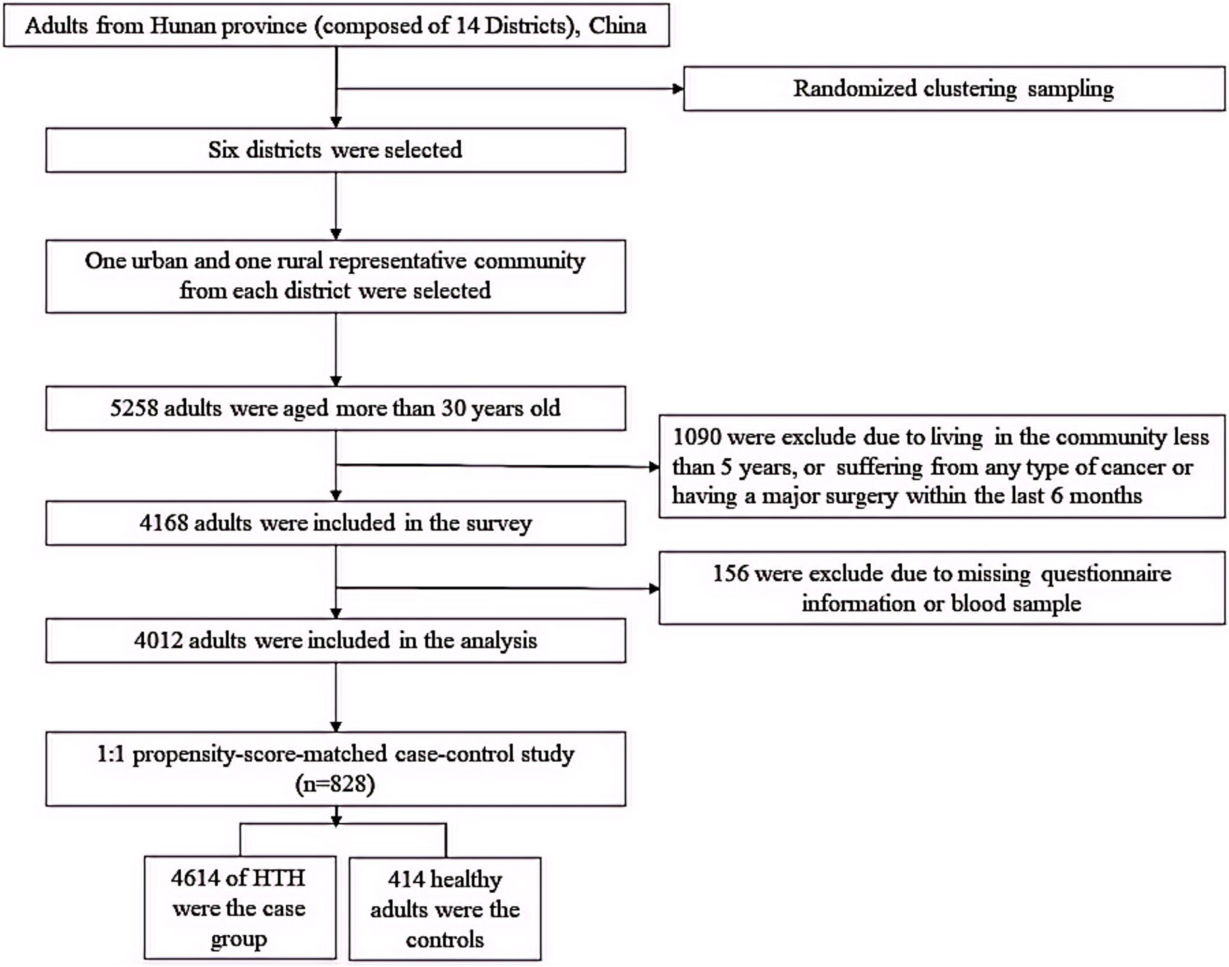
Flowchart of the study.

In this study, firstly, we conducted a population-based cross-sectional study to explore the association of Hcy with CMM, then, a 1:1 matched case–control study was conducted using propensity-score-matched method to validate this association. A representative sample was obtained by multistage cluster random sampling design from July 2013 to March 2014 in Hunan Province (including 14 districts and a population of more than 66 million), China. Detailed information on study design of this population has been described in our previous study (16). Those met the inclusion and exclusion criteria signed written informed consent. After obtaining informed consent, eligible participants were asked to complete a questionnaire. Accordingly, 4,012 participants were included in the population-based cross-sectional study to explore the relationship between Hcy and CMM. Furtherly, a 1:1 matched case–control study based on the propensity-score-matched method (414 No-CMM and 414 CMM) was conducted to verify this effect (Figure 1). The matched factors include age, sex, educational attainment, family income, occupation status, marital status, current smoking, heavy alcohol consumption, unhealthy diet, inactive exercise, sedentary behavior, BMI, WC, TC, TG, LDL-C and HDL-C because each factor individually contributes to increased risk of cardiometabolic diseases according to previous researches (16, 17).

The sample size estimation formula for cross-sectional studies was as follow: $n=\frac{{u_{\alpha }}^{2}p\left(1-p\right)}{\delta^{2}}$ , here, *δ* and *p* represents the estimated allowable error and population rate *π*, respectively. According to the previous study, the prevalence of HHcy in the population is 35.4% (18), with an error requirement of no more than 3%, if We assume *α* = 0.05, then, the sample size obtained is 977. A total of 4,012 subjects were included in this study, which meet sample size needs. The test power was 1.000 based on the prevalence of HHcy (58.3and 32.2%, respectively) in the CMM group (436 participants) and non-CMM group (3,576 participants). In addition, the sample size estimation formula for 1:1 case–control study was as follows: $m=\frac{{\left[Z_{1-a/2}/2+Z_{\beta }\sqrt{P\left(1-P\right)}\right]}^{2}}{{\left(P-0.5\right)}^{2}}$ , here, *m* refers to the number of pairs with inconsistent exposure status between the case and control, and $P=\mathrm{OR}/\left(1+\mathrm{OR}\right)$ . Next, calculate the total number of required pairs (M): $M=\frac{m}{P_{0}\left(1-P_{1}\right)+P_{1}\left(1-P_{0}\right)}$ , here, *P_0_* and *P_1_* are estimated exposure rates for the control group and the case group, respectively. Based on previous literature reports, the HHcy exposure rate in the control group was 66.4%, OR = 2.08 (19), and the setting *α* = 0.05 (bilateral), *β* = 0.20, and the final estimated sample size was 127 pairs. The case and control groups in this study included 414, respectively, which meet the sample size needs. The test power was 1.000 based on the prevalence of HHcy in the case and control groups (57.2 and 36.7%, respectively).

### Data collection

Well-trained investigators collected data on socioeconomic characteristics through face-to-face and one-to-one questionnaires, which was referred the questionnaire of the China Health and Retirement Longitudinal Study (CHARLS) (20), including demographic characteristics (including age, sex, education, family income, occupation status and marital status) and lifestyles factor (including smoking, alcohol drinking, exercise and diet). Here, a test–retest reliability test was performed on the questionnaire, with a Cronbach’s *α* coefficient in this sample being 0.778. Education level: below of high school, ordinary/vocational high school and undergraduate/college degree. Family income per year was asked for every participant, and further divided into three groups: low, medium and high. Marital status groups: unmarried, married/cohabitation and divorce/widow. Occupation types: wage-laborer, white-collar worker, farmer and retiree. Smoking was defined in the questionnaire as smoking more than 100 cigarettes in life. The drink frequency (days) and amounts (drinks) in the past week were reported according to a set of simple and easy-to-understand photos to measure the drinks of different kinds of drinks (see in Supplementary Figure S1). The average daily alcohol drinks were estimated as follows: average daily alcohol drinks = (frequency [days] × (amounts [drinks] in each of those days))/7 (18). Heavy alcohol consumption level was defined as average daily alcohol drinks ≥2. We evaluated dietary status using food frequency questionnaire according to a more recent dietary recommendation for blood pressure and combining with traditional Chinese eating habits, which considered adequate consumption of fresh fruit, fresh vegetables, unprocessed meats (including red meat, fish or shellfish), reduced consumption of high-fat, high-salt and sugar-sweetened food. We defined an unhealthy diet as meeting less than four items of the recommendations (see Supplementary methods for details). For physical activity, the number of days per week that the participants did physical activities (such as weightlifting, stair climbing, fast cycling, aerobics, running, etc.) and the time of each exercise were obtained through the questionnaires. Inactive exercise was defined as having lasting for less than 10 continuous minutes per week (21). Sedentary behavior was defined as sitting still for more than 3 hours per day.

### Anthropometric measurement

Systolic and diastolic blood pressure was measured by professionally trained staff using the calibrated electronic automatic tester. Each participant had their blood pressure measured three times with at least 5 min of rest each time. The average of the 3 values was calculated and documented as the final blood pressure value. Weight, height, and waist circumference (WC) were measured by trained staff using well-calibrated instruments. Body-mass index (BMI) was calculated as bodyweight in kg divided by the square of height in meters, and a value more than or equal to 24 was defined as an abnormal BMI.

### Biochemical measurement

Blood samples were collected at 07:30–10:00 after a fasting period of 12 h. The plasma Hcy was measured by trained laboratory technicians using the microplate enzyme immunoassay method, with homocysteine Detection Kit of MedicalSystem Biotechnology Co., Ningbo, China (Reagent batch number, 13082408). Other laboratory indicators included fasting blood-glucose (FPG), plasma total cholesterol (TC), triglyceride (TG), low density lipoprotein cholesterol (LDL-C), high density lipoprotein cholesterol (HDL-C) and C-reactive protein (CRP) were detected using a Hitachi 7,600 Automatic Biochemistry Analyzer (Hitachi).

### Definition of cardiometabolic diseases

Among cardiometabolic diseases, we focus on the diabetes, hypertension, coronary heart disease, and stroke. Diabetes mellitus was defined as a fasting blood glucose level ≥ 7.0 μmol/L or clinically diagnosed with diabetes mellitus or taking hypoglycemic drugs. Hypertension was defined as a diastolic/systolic blood pressure ≥ 90/140 mm/Hg or clinically diagnosed with hypertension or taking antihypertensive drugs. Coronary heart disease and stroke could be defined as a self-reported disease diagnosed by a physician or hospitalization record. Cardiometabolic multimorbidity was defined as the coexistence of two or three cardiometabolic diseases, including hypertension, diabetes, coronary heart disease and stroke.

### Statistical analysis

The mean and standard deviation were used to describe the normal distribution of quantitative variables, and the *t* test was used to compare the differences. The median and quartile intervals were used to represent the quantitative variables that did not follow the normal distribution, and the *Wilcoxon rank sum* test was used to compare the differences. Categorical variables were expressed as counts and percentages (%), and differences were compared using *Chi-square* test or *Fisher exact probability* method.

Participants were categorized into two groups based on the number of CMM: ≥2 and none. Quartile classifications of Hcy level was generated (first quartile [Q1]: < 10.4 μmol/L, second quartile[Q2]: 10.4 ~ 13.2 μmol/L, third quartile[Q3]:13.3 ~ 16.2 μmol/L, fourth quartile [Q4]: >16.2 μmol/L). Logistic regression models were employed to evaluate the associations between Hcy and CMM using odds ratios (ORs) and 95% confidence intervals (CIs). The potential non-linear relationships of Hcy with CMM was estimated by using a restricted cubic spline (RCS) model. “VennDiagram” and “openxlsx” R packages were used to implement multimorbidity pattern analysis.

Here, to validate the association of Hcy with CMM, a 1:1 matched case–control study was established using propensity score matching method. The data before and after matching were, respectively, compared to see whether the patients were balanced in important covariates. The difference of the covariates after matching was greater than 0.06, indicating that the covariates after matching were balanced among groups (see Supplementary Table S1 for details). In this case–control study, to examine the proportion of CMM in the exposed population that theoretically would not have occurred if all participants had adhered to the lowest level of Hcy (Q1), we calculated the attributable risk proportion (ARP) under the assumption of a causal relationship between the highest level of Hcy (Q4) and CMM risk. The formula for calculating ARP was as follows: ARP = (OR−1)/OR×100%.

To identify the potential mechanism for the association, we conducted a mediation effect model as Hcy (X) → biomarker (M) → CMM(Y). A biomarker was selected if it was significantly correlated with both Hcy and CMM. Detailly, a logistic model was used when CMM was the dependent variable, and a linear model was used when the biomarker was the dependent variable. Bias-corrected *bootstrap* method with 1,000 resamples was used to obtain 95% CIs of the direct and indirect effects (22). Age, sex, educational attainment, family income, occupation status, marital status, current smoking, heavy alcohol consumption, unhealthy diet, inactive exercise and sedentary behavior were adjusted in mediation models as covariates. Coefficients are presented in standardized form, using standardized coefficients as indices of effect. A statistically significant mediation effect is observed when the 95%CI does not include zero. The mediated proportion was used to evaluate the effect size of the mediation analysis.

To assess the robustness of the results, four models were constructed in the sensitivity analysis based on previous researches (16, 18). In model 1, no covariate was adjusted. In model 2, we adjusted for age, sex, education, family income, marital status and occupational status. In model 3, sedentary behavior should be added as an adjusted variable. In model 4, additionally adjusted for serum FPG, TC, TG, LDL-C and HDL-C and CRP based on Model 3. In addition, subgroup analyses were performed according to different combinations of CMMs.

All statistical analyses were conducted using STATA version 18.0 (Institute, Gary, NC, United States) and R version 4.4.1. A two-sided *p* < 0.05 was considered statistically significant.

## Results

### Population characteristics

A total of 4,012 participants were enrolled in this study for analysis (Table 1). The study population had a mean age of 54.6 years (SD 12.6). 1,644 (41.0%) were males and 2,368 (59.0%) were females. Among them, 2,179 (54.3%) of 4,012 individuals did not have a CMD, whereas 1833 (45.7%) had at least one of these diseases. The prevalence rate of diabetes, hypertension, CHD and stroke were 12.2% (491/4012), 34.8% (1,394/4012), 7.7% (310/4012), and 2.8% (114/4012), respectively. The prevalence of CMM was 10.9% (436/4012). Compared with people who did not have CMM, those with CMM were more likely to be older, male, have low educational attainment and family income, be farmers or retirees, be currently smoking, heavy drinking, unhealthy in diet, sedentary and overweight/obesity, and have diabetes, hypertension, stroke and CHD (Table 1). The CMM group had elevated mean levels of WC, FPG, SBP, DBP, TG, TC and CRP (88.9 ± 10.6, 7.5 ± 2.4, 153.7 ± 20.5, 87.5 ± 13.7, 2.4 ± 2.0, 4.9 ± 1.0, and 6.1 ± 2.4 mmol/L, respectively) than the No-CMM group. The prevalence rate of diabetes, hypertension, CHD and stroke were 12.2% (491/4012), 34.8% (1,394/4012), 7.7% (310/4012), and 2.8% (114/4012), respectively. Notably, those with CMM were more likely to have high level of plasma Hcy than population with No-CMM.

**Table 1:** Baseline characteristics of participants by cardiometabolic disease status^a^.

Characteristics	Total	No-CMM	CMM	*p*-value
*N*	4,012	3,576	436	
Age, mean (SD)	54.6 (12.6)	53.34 (12.3)	64.7 (10.4)	<0.001
Sex (%)				<0.001
Male	1,644 (40.1)	1,408 (39.4)	236 (54.1)	
Female	2,368 (59.0)	2,168 (60.6)	200 (45.9)	
Educational attainment (%)				<0.001
Below of High School	2033 (50.7)	1726 (48.3)	307 (70.4)	
Ordinary or vocational high school	1,154 (28.7)	1,058 (29.6)	96 (22.0)	
Undergraduate or college degree	825 (20.6)	792 (22.1)	33 (7.6)	
Family income^b^ (%)				<0.001
Low	1,159 (38.8)	1,348 (37.7)	211 (48.4)	
Medium	1,439 (35.9)	1,286 (36.0)	153 (35.1)	
High	1,014 (25.3)	942 (26.3)	72 (16.5)	
Occupation (%)				<0.001
Wage laborer	595 (14.8)	555 (15.5)	40 (9.2)	
White-collar worker	1,102 (27.5)	1,064 (29.8)	38 (8.7)	
Farmer	714 (17.8)	624 (17.4)	90 (20.6)	
Retiree	1,601 (39.9)	1,333 (37.3)	268 (61.5)	
Marital status (%)				0.057
Unmarried	65 (1.6)	59 (1.6)	6 (1.4)	
Married/Cohabitation	3,808 (94.9)	3,384 (94.6)	424 (97.2)	
Divorce/Widow	139 (3.5)	133 (3.7)	6 (1.4)	
Current smoking (%)	1,079 (26.9)	904 (25.3)	175 (40.1)	<0.001
Heavy alcohol drinking (%)	962 (24.0)	837 (23.4)	125 (28.7)	0.017
Unhealthy diet (%)	1974 (49.2)	2,308 (64.5)	347 (79.6)	<0.001
Inactive exercise (%)	2,610 (65.1)	2,319 (64.8)	291 (66.7)	0.460
Sedentary behavior (%)	2,333 (58.2)	2060 (57.6)	273 (62.6)	0.045
BMI ≥24Kg/m^2^ (%)	1,578 (39.3)	1,347 (37.7)	231 (53.0)	<0.001
WC, mean (SD)	84.4 (9.9)	83.8 (9.6)	88.9 (10.6)	<0.001
DBP, mean (SD)	127.4 (21.0)	78.7 (11.4)	87.5 (13.7)	<0.001
SBP, mean (SD)	79.6 (12.0)	124.2 (18.6)	153.7 (20.5)	<0.001
FPG, mean (SD)	5.7 (1.6)	5.4 (1.3)	7.5 (2.4)	<0.001
TC, mean (SD)	4.8 (0.9)	4.8 (0.9)	4.9 (1.0)	0.013
TG, mean (SD)	2.0 (16)	1.9 (1.6)	2.4 (2.0)	<0.001
LDL-C, mean (SD)	2.6 (0.8)	2.6 (0.8)	2.6 (0.8)	0.056
HDL-C, mean (SD)	1.3 (0.3)	1.3 (0.3)	1.3 (0.4)	0.970
CRP, mean (SD)	5.5 (1.6)	5.4 (1.4)	6.1 (2.3)	<0.001
HCY, mean (SD)	13.8 (7.7)	13.5 (7.9)	15.9 (4.5)	<0.001
Diabetes (%)	491 (12.2)	227 (6.3)	264 (60.6)	<0.001
Hypertension (%)	1,394 (34.8)	994 (27.8)	400 (91.7)	<0.001
Stroke (%)	114 (2.8)	42 (1.2)	72 (16.5)	<0.001
CHD (%)	310 (7.7)	134 (3.7)	176 (40.4)	<0.001
CMM (%)				<0.001
0	2,179 (54.3)	2,179 (60.9)	0 (0.0)	
1	1,397 (39.1)	1,397 (39.1)	0 (0.0)	
≥2	436 (10.9)	0 (0.0)	436 (100.0)	

CMM = cardiometabolic comorbidities, CHD = coronary heart disease, SD = standard deviation; BMI = body mass index; WC = waist circumference; FPG = fasting blood-glucose, TC = total cholesterol, TG = triglyceride, LDL-C = low density lipoprotein cholesterol, HDL-C = high density lipoprotein cholesterol, CRP = C-reactive protein, HCY = Homocysteine.

a Continuous variables were expressed as mean (standard deviation), and categorical variables were expressed as number (percentage). *p-values* were calculated using analysis of variance for continuous variables, and Pearson chi-squared test or Fisher’s exact for categorical variables.

b Family income was divided into high, medium, and low level according to triquartile method.

### Multimorbidity pattern analysis

**Figure 2:**
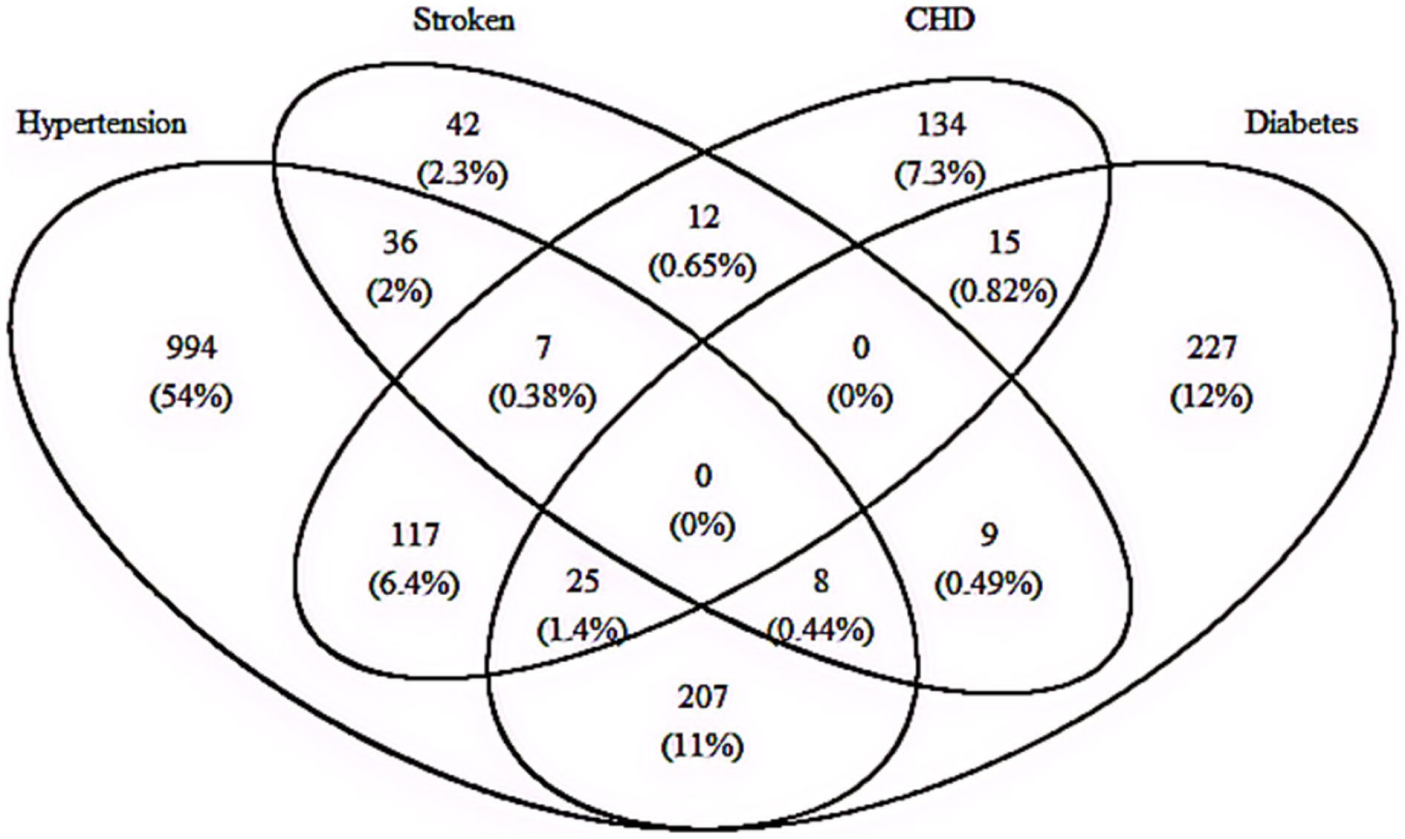
Multimorbidity pattern analysis. CHD, coronary heart disease.

Figure 2 provides details about the combinations of different cardiometabolic diseases and includes 15 combinations in total. Among the 4,102 participants enrolled in this study, 1833 had at least one of CMD (45.7%) and 436 (10.9%) met the diagnostic criteria for CMM, which includes metabolic disorders such as hypertension, diabetes mellitus, stroke and CHD. Of those with CMM, the most two frequent combinations were the “Diabetes + Hypertension” and “Hypertension + CHD,” corresponding to 47.5% (207/436) and 26.8% (117/436), respectively. Additionally, among the total study participants 9.2% (40/436) had there or more component conditions of CMM (Figure 2). Here, the associations of plasma Hcy with the risk of specific combination of CMD were displayed in Supplementary Table S2. After adjusting for age, sex, educational attainment, family income, occupation status, marital status, current smoking, heavy alcohol consumption, unhealthy diet, inactive exercise, sedentary behavior, BMI, WC, TC, TG, LDL-C, HDL-C and CRP, we found those with diabetes, hypertension and CHD had corresponding ORs of 1.97 (95% CI: 1.15–3.56, *p* = 0.013), 11.35 (95% CI: 5.58–23.09, *p* < 0.001), and 1.98 (95% CI: 1.05–3.74, *p* = 0.034), respectively. No significant association was found between plasma Hcy and stroke (OR = 1.00, 95% CI: 0.92–1.05, *p* = 0.691).

### Associations of homocysteine with cardiometabolic multimorbidity and its ARP

**Figure 3:**
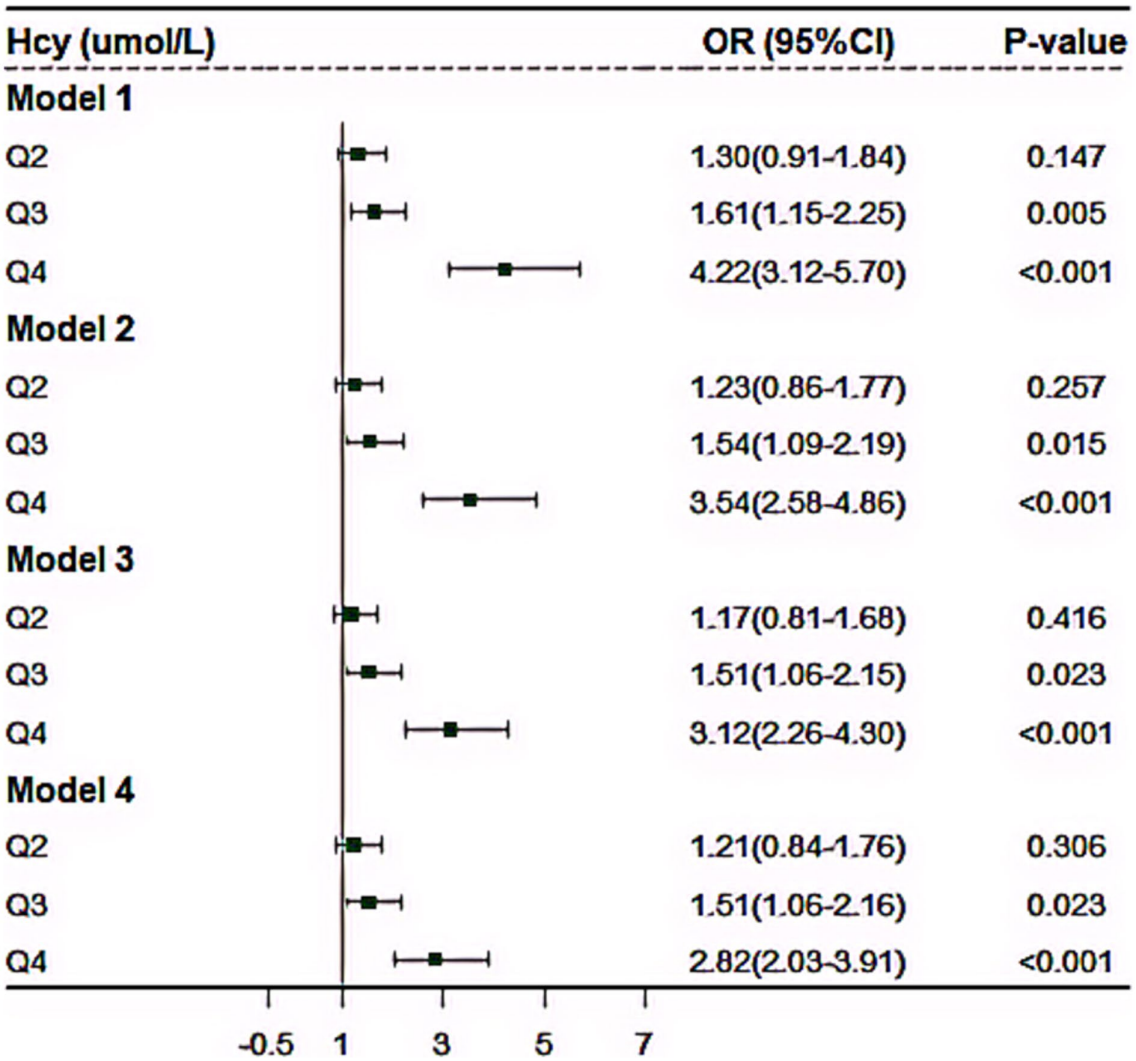
The effect of plasma homocysteine levels on cardiometabolic multimorbidity based on 4,012 community population. Model 1, no covariate was adjusted; Model 2, we adjusted for age, sex, education, family income, marital status and occupational status; Model 3, as further adjusted for current smoking, heavy drinking, unhealthy diet, inactive exercise and abnormal BMI based on Model 2; Model 4, additionally adjusted for serum FPG, TC, TG, LDL-C and HDL-C, and CRP based on Model 3.

**Figure 4:**
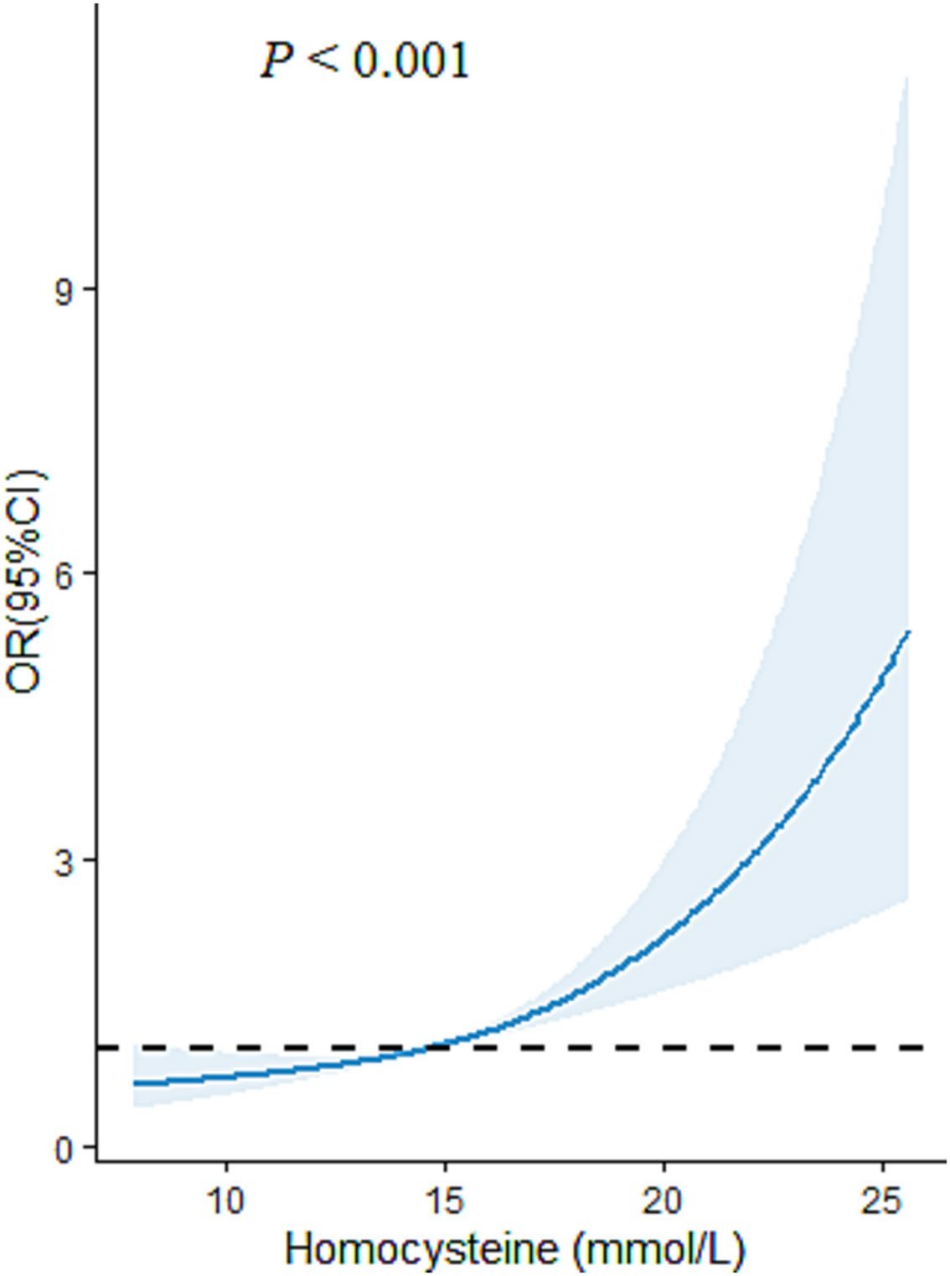
The restricted cubic spline (RCS) model for the relationship of plasma Hcy level with CMM in Chinses adults. The reference value for Hcy was set as a cut-off value for the first quartile. Three nodes were selected for all models. All models were adjusted for age, sex, educational attainment, family income, marital status, occupational status, smoking, heavy alcohol consumption, unhealthy diet, inactive exercise, sedentary behavior.

Multiple logistic regression models were carried out to examine the impact of plasma level of Hcy on the risk of CMM (Figure 3). In the cross-sectional study based on 4,012 participants, after adjusted for the covariables (including age, sex, educational attainment, family income, occupation status, marital status, current smoking, heavy alcohol consumption, unhealthy diet, inactive exercise, sedentary behavior, BMI, WC, TC, TG, LDL-C, HDL-C and CRP), compared with the lowest level of plasma Hcy [Q1], the third (OR = 1.51, 95%CI:1.06–2.16, *p* = 0.023) and fourth quartile levels [Q4] (OR = 2.82, 95%CI: 2.03–3.91, *p* < 0.001) of Hcy were significantly and positively associated with increased CMM risk. Moreover, RCS analysis showed that there was an inverse dose–response relationship between plasma Hcy concentration and CMM after adjusted for the covariables, suggesting that escalating levels of Hcy was associated with an increasing risk of developing CMM (*p* < 0.001) (Figure 4).

In the propensity-score-matched case–control study (414 non-CMM vs. 414 CMM), 828 participants were included. We validated the results of the above cross-sectional study that plasma Hcy had positive impact on risk of CMM (Table 2). For details, compared with those with lowest quartile level of Hcy, the risk of CMM was increased by 183% (OR = 2.83, 95% CI: 1.84–4.36, *p* < 0.001) among those with highest fourth quartile level of Hcy. Table 2 also summarized the ARP for plasma level of Hcy. In case–control study, the multivariate ARP of highest fourth quartile level of Hcy was 64.66% (95% CI: 46.24–77.06%).

**Table 2:** The effect of plasma homocysteine levels on cardiometabolic multimorbidity and its attribution risk percentage based on 1:1 matching case–control study^a^.

Homocysteine (mmol/L)	NO-CMM *vs.* CMM		
	OR	95%CI	*p*-value
Q1	1.00		
Q2	1.16	0.73–1.84	0.535
Q3	1.50	0.95–2.38	0.083
Q4	2.83	1.84–4.36	<0.001
Trend for per unit	1.12	1.08–1.16	<0.001
ARP (%, 95CI)	64.66	46.24–77.06	

a Adjusted for age, sex, educational attainment, family income, occupation status, marital status, current smoking, heavy alcohol consumption, unhealthy diet, inactive exercise, sedentary behavior, BMI, WC, TC, TG, LDL-C and HDL-C.

### Mediation analysis

Supplementary Tables S3, S4 illustrated the correlations of plasma Hcy with various potential mediated indicators and the association of potential mediated indicators with CMM. Since CRP, TC, TG and WC were significantly correlated with both Hcy and CMM, we hypothesis that these indicators may mediate the association we investigated. We performed a causal mediation analysis, and recognized a proportion of effect mediated by CRP, TC, TG and WC as 7.77% (95% CI: 2.87–16.00%, *p* < 0.001), 12.00% (95% CI: 5.10–21.00%, *p* < 0.001), 18.93% (95% CI: 8.96–40.00%, *p* < 0.001) and 22.58% (95% CI: 11.26–56.00%, *p* < 0.001), respectively (Supplementary Figure S2).

### Sensitivity and subgroup analysis

In sensitivity analyses, we constructed several models, then, we found the results remained similar in all sensitivity analyses (Figure 3). Moreover, we conducted subgroup analysis. Supplementary Figure S3 showed the results stratified by different combinations of CMM. For details, the plasma level of Hcy was mostly correlated with the combination of diabetes, hypertension and CHD (OR = 2.26, 95%CI: 1.43–3.57, *p* < 0.001), followed by the combination of diabetes and hypertension (OR = 1.34, 95%CI: 1.23–1.44, *p* < 0.001), the combination of hypertension and CHD (OR = 1.32, 95%CI: 1.21–1.44, *p* < 0.001) and the combination of hypertension and stroke (OR = 1.19, 95%CI: 1.04–1.37, *p* = 0.013), respectively (Supplementary Figure S3).

## Discussion

In this community-based study, we investigated the association of Hcy with CMM (two or more conditions in diabetes, hypertension, coronary heart disease, and stroke) in a group of adults over 30 years of age in China, with the largest effect combination of CMM being diabetes, hypertension and coronary heart disease. An inverse association and dose–response relationship were observed between CMM and plasma Hcy levels. Highest level of plasma Hcy (> 16.2 μmol/L) explained about 64.66% of the CMM risk. We also recognized a significant mediation effect by C-reactive protein, total cholesterol, triglyceride and waist circumference in associations of Hcy with CMM, with corresponding mediation proportion ranging from 8 to 23%. Our findings suggested a targeted approach to dynamically monitor and reduce plasma Hcy levels to reduce the risk of cardiometabolic multimorbidity.

Hcy was identified by Butz et al. (23), as an important intermediate product in the methionine cycle and cysteine metabolism. Subsequently, an 8-year-old intellectually disabled boy died strangely from a severe heart attack, and HHcy’s description was first reported. Since that time, the number of clinical and basic studies of Hcy has increased exponentially. In certain situation, the risks associated with HHcy may be significantly elevated. There is evidence that HHcy is a high predictive value for diabetes patients (24), hypertension (25), and cardiovascular and cerebrovascular diseases (26). In our study, we found high level of plasma Hcy was positively related to elevated risk of diabetes, supporting the result from other study (24). In addition, Hcy was also associated with increased risk of the occurrence of complications of diabetes, such as diabetic nephropathy, diabetic retinopathy, etc. In the future, Hcy can potentially be used as an early screening indicator for diabetic microvascular complications (27). Additionally, homocysteine, as one of one-carbon metabolism components, is also closely entwined with gestational diabetes mellitus risk (28). Therefore, it is very important to understand the origin and metabolism of homocysteine and the pathway of its change in diabetes pathogenesis.

In 2007, a study on the efficacy and safety of enalapril folic acid tablets in lowering blood pressure and plasma Hcy was conducted in six major cities in China (6). Thus, Chinese researchers first proposed the concept of HTH, and found elevated Hcy levels were associated with 75% of the hypertension cases (7). Numerous human epidemiological studies have reported the relationship between plasma Hcy levels and hypertension different populations (25, 29). In our study, we found those with over 16.2 umol/L plasma Hcy level had a 11.35 times higher risk of hypertension than those in lowest levels (<10.2 umol/L), which was consistent with serval studies (25, 29, 30). Notably, HTH was significantly amplifying the damage of hypertension to blood vessels, increasing the risk of heart, brain, and kidney complications, and increasing the risk of cardiovascular events, especially stroke (9).

Since the observation, by McCully et al., of severe arteriosclerotic lesions in children with high level of plasma Hcy, a numerous series of observational studies have demonstrated elevation of plasma Hcy level was considered be related to cardiovascular disease (CVD), stroke, and venous thromboembolism (31, 32). Another study suggested that even slight increases in plasma Hcy levels can enhance the risk of CVD (33). In our study, we recognize the high level of plasma Hcy (> 16.2 μmol/L) plays a key role in prevalence to coronary heart disease (OR = 1.98, *p* = 0.034). A review reported that primary stroke can at least in part be prevented by lowering total homocysteine. Detailly, total Hcy values in adults of 10 μmol/L or below are probably safe, but that values of 11 μmol/L or above may justify intervention (34). The strongest evidence that Hcy plays a causal role in atherothrombosis reducing stroke has been provided by studies using animal models (35). These studies support the role of Hcy in the pathogenesis of atherosclerosis, suggesting that Hcy may be a disease biomarker (34). However, some studies have proposed different views on the relationship between Hcy and stroke. Recent meta-analysis did not show that reducing Hcy treatment was associated with incidence rate of all-cause death or coronary artery disease, except for prevention for stroke (36). Therefore, it is still uncertain that whether Hcy is a causative factor or a biomarker of vascular disease in human beings. Further research may be needed to elucidate its causal role and mechanism on vascular disease in different populations.

With the aging of the global population and the development of medical technology, the number of chronic diseases is constantly expanding, and more and more chronic diseases are being controlled at a certain level, resulting in delayed survival time for patients (37). This has led to an increasing number of people suffering from CMM (co-occurrence of at least two CMDs, including diabetes, hypertension, stroke and coronary heart disease), posing a huge challenge to the healthcare system (38). It has been confirmed that plasma Hcy is highly correlated with individual CMDs (such as diabetes, hypertension and coronary heart disease), as shown by our present findings, to the best of our knowledge, however, no research has further evaluated the impact of plasma HCY on CMM, except for one research that reported negative result (10). Our study provides important and innovative results to explore the strong correlation between plasma Hcy and CMM, and dose–response relationship analysis suggests that the higher the plasma Hcy level, the greater the risk of CMM. And, in our sensitivity analysis, the results were not materially changed with those of the main analyses. Notably, our subgroup analysis showed that high level of plasma Hcy was most associated with the combination of coexistence of diabetes, hypertension and CHD among the general population in China. Critically, we calculate the ARP for exposure on high level of plasma Hcy. In case–control study, the multivariate ARP of highest fourth quartile level of Hcy was 64.66% (95% CI: 46.24–77.06%). It means that the percentage of CMM attributable to Hcy in those exposed to the highest levels of plasma Hcy (> 16.2 umol/L) was 64.66%, indicating that 64.66% of those with high levels of Hcy would avoid CMM if they reduced their Hcy levels to the lowest one.

Cardiometabolic disease spectrum has gradually expanded to any metabolic disease that can affect the function and/or structure of the heart, including diabetes, CVD, hypertension, chronic renal insufficiency, etc., forming a chronic disease spectrum characterized by metabolic disorder of multiple organ systems (39). In terms of mechanism, with the continuous deepening of relevant clinical and basic research, the academic community has found that a series of pathological and physiological changes, such as inflammation, glucose and lipid metabolism disorders, oxidative stress, hormone regulation imbalance, and changes in intracellular and extracellular signaling pathways, are involved in the pathogenesis of CMM (40). Among them, inflammation damage caused by macrophages which typically exhibit classic M1 polarization, then secrete pro-inflammatory cytokines, participate in inflammatory responses (such as CRP, IL-6, etc.), and promote vascular damage, playing a core role in the development of CMD (41, 42). In other hand, lipid metabolism disorder caused by excessive oxidative stress participates in the pathogenesis of CMD (40). Similarly, data from UK Biobank found that evidence of serum TC and TG are causally related to the risks of cardiometabolic multimorbidity (43). In our study, cellular inflammatory factor (CRP) and abnormal indicators of lipid metabolism (TC, TG and WC) mediate a small proportion (approximately 8 ~ 23%) of the association of Hcy with CMM, suggesting that the unexplained variations might be attributable to other mechanisms of CMM. CRP and lipids indicators (TC, TG) may serve as early biomarkers of Hcy-related CMM. However, more investigations are warranted to target the clinical heterogeneity of CMM.

This study we conducted has some strengths. To the best of our knowledge, our study provides important and new evidence to this field that those with plasma Hcy levels exceeding 16.2 umol/L have a higher risk of CMM, and confirmed that Hcy was mostly associated with the combination of diabetes, hypertension and CHD, implicating for cardiologists and decision makers that CMM can be attributable to high level of plasma Hcy and it has become a public threat persistently affecting cardiovascular health in humans. Furthermore, we use a PSM case–control study to verify the positive association between Hcy and CMM found in cross-sectional study, which can enhance the objectivity of our results, using similar covariate distributions to construct case and control groups without affecting the results of the study (12). As a semi-parametric method, PSM was an important set of tools for estimating causes of disease or treatment effects in observational studies, enabling adjustment for measured confounders in an easy-to-understand and transparent way (12). However, there were still several potential limitations that should be considered when interpretating the results. Firstly, the plasma Hcy level was only assessed by at only one-time point, which could not successfully reflect the true dynamic levels of individual Hcy exposure. Future studies with repeated assessment are very important and indispensable. Besides, participants in current study are from Hunan province, China, and the sample of this special population is considered large, however, the generalizability of our results may be constrained in other regions in China and in other countries. Therefore, multiprovince or multinational monitoring studies will be necessary. Additionally, some variables, like lifestyle factors, were self-reported and only evaluated at a point in time, and it was not possible to completely avoid recall or evaluation biases. Moreover, due to the cross-sectional and case–control design of this study, causality cannot be inferred. Therefore, further larger prospective investigations are required to confirm our results. Finally, the associations between Hcy and single CMDs (hypertension, diabetes and CHD) are well-established, however, a limited number of studies examined the associations between Hcy and CMM. Our study provides important and new evidence to this fields, however, it still needs to be validated in other additional studies in the future.

In conclusion, our findings add new evidence to this field that of high level of plasma Hcy is consistently associated with higher risk of CMM among Chinses adults, with the largest effect combination of coexistence of diabetes, hypertension and coronary heart disease. These findings have implications for cardiologists that CMM can be attributable to high level of plasma Hcy, and for decision makers that Hcy has become a public threat that persistently affects cardiovascular health in humans. There is an urgent need for a targeted approach to dynamically monitor and reduce plasma Hcy levels to reduce the risk of CMM.

## Data Availability

The original contributions presented in the study are included in the article/Supplementary material, further inquiries can be directed to the corresponding author.
