# Supplementary material for: Effect of plasma homocysteine on cardiometabolic multimorbidity among Chinese adults: a population-based and real-world evidence study
Source: Front Nutr. 2024 Dec 20;11:1522212. doi: 10.3389/fnut.2024.1522212 (PMC11695421; doi:10.3389/fnut.2024.1522212)
Supplement: Supplementary file 1 [file Table_1.DOCX]

**Supplementary methods of assessment of unhealthy diet.**

We evaluated dietary status using a more recent dietary recommendation for cardiovascular disease and combining with traditional Chinese eating habits, which considered adequate consumption of fresh fruit, fresh vegetables, unprocessed meats (including red meat, fish or shellfish), reduced consumption of high-fat, salted fish/meat and sugar-sweetened food. The unhealthy diet was defined as at least 4 of the healthy food items.

**Table 1 for supplementary method**. Components of more recent dietary recommendations for blood pressure health.

| **Diet complement** | **Intake goal** |
| --- | --- |
| Fresh vegetable | Consumption every day |
| Fresh fruit | Consumption every day |
| Unprocessed meats | More than two times/week |
| High-fat diet | ≤ Once a week |
| Pickled fish or meat | ≤ Once a week |
| Sugar-sweetened food | No consumption |

**Table S1**. Distribution of characteristics among CMM and No-CMM.

|  |  | **Overall data** | | | | **Propensity 1:1 Matching** | | |
| --- | --- | --- | --- | --- | --- | --- | --- | --- |
| **Variables** |  | **Control**  **(n=3576)** | | **Case**  **(n=436)** | ***p-value*** | **Control**  **(n=414)** | **Case**  **(n=414)** | ***p-value*** |
| Region |  |  | |  | <0.001 |  |  | 0.292 |
| Changsha |  | 1831(51.2) | | 187(42.9) |  | 202(48.8) | 173(41.8) |  |
| Zhuzhou |  | 637(17.8) | | 127(29.1) |  | 108(26.1) | 123(29.7) |  |
| Hengyang |  | 489(13.7) | | 53(12.2) |  | 37(8.9) | 51(12.3) |  |
| Yueyang |  | 392(10.9) | | 54(12.4) |  | 48(11.6) | 52(12.6) |  |
| Yongzhou |  | 128(3.6) | | 8(1.8) |  | 11(2.7) | 8(1.9) |  |
| Xiangxi |  | 99(2.8) | | 7(1.6) |  | 8(1.9) | 7(1.7) |  |
| Age (years, SD) |  | 53.34(12.3) | | 64.7(10.4) | <0.001 | 64.9(10.9) | 64.7(10.5) | 0.249 |
| Sex |  |  | |  | <0.001 |  |  | 0.889 |
| Male |  | 1408(39.4) | | 236(54.1) |  | 226 (54.6) | 224 (54.1) |  |
| Female |  | 2168(60.6) | | 200(45.9) |  | 188 (45.4) | 190 (45.9) |  |
| Family income |  |  | |  | <0.001 |  |  | 0.142 |
| Low |  | 1348(37.7) | | 211(48.4) |  | 217 (52.4) | 195 (47.1) |  |
| Medium |  | 1286(36.0) | | 153(35.1) |  | 137 (33.1) | 150 (36.2) |  |
| High |  | 942(26.3) | | 72(16.5) |  | 60 (14.5) | 69 (16.7) |  |
| Educational attainment |  |  | |  | <0.001 |  |  | 0.103 |
| Below of High School | | | 1726(48.3) | 307(70.4) |  | 312 (75.4) | 286 (69.1) |  |
| Ordinary/Vocational high school |  | 1058(29.6) | | 96(22.0) |  | 72 (17.4) | 95 (22.9) |  |
| Undergraduate/college degree |  | 792(22.1) | | 33(7.6) |  | 30 (7.2) | 33 (8.0) |  |
| Marital status |  |  | |  | 0.057 |  |  | 0.869 |
| Unmarried |  | 59(1.6) | | 6(1.4) |  | 6 (1.4) | 3 (0.7) |  |
| Married/Cohabitation |  | 3384(94.6) | | 424(97.2) |  | 401 (96.9) | 405 (97.8) |  |
| Divorce/Widow |  | 133(3.7) | | 6(1.4) |  | 7 (1.7) | 6 (1.5) |  |
| Occupation status |  |  | |  | <0.001 |  |  | 0.654 |
| Wage-labourer |  | 555(15.5) | | 40(9.2) |  | 27 (6.5) | 37 (8.9) |  |
| White-collar worker |  | 1064(29.8) | | 38(8.7) |  | 37 (8.9) | 37 (8.9) |  |
| Farmer |  | 624(17.4) | | 90(20.6) |  | 127 (30.7) | 85 (20.5) |  |
| Retiree |  | 1333(37.3) | | 268(61.5) |  | 223 (53.9) | 255 (61.6) |  |
| Current smoking (%) |  | 904(25.3) | | 175(40.1) | <0.001 | 171 (41.3) | 161 (38.9) | 0.479 |
| Heavy alcohol drinking (%) |  | 837(23.4) | | 125(28.7) | 0.017 | 122 (29.5) | 115 (27.8) | 0.591 |
| Unhealthy diet (%) |  | 2308(64.5) | | 347(79.6) | <0.001 | 325 (78.5) | 325 (78.5) | 1.000 |
| Inactive exercise (%) |  | 2319(64.8) | | 291(66.7) | 0.460 | 285 (68.8) | 278 (67.1) | 0.603 |
| Sedentary behavior (%) |  | 2060(57.6) | | 273(62.6) | 0.045 | 383 (92.5) | 392 (94.7) | 0.202 |
| BMI ≥24Kg/m^2^ (%) |  | 1347(37.7) | | 231(53.0) | <0.001 | 184 (44.4) | 213 (51.4) | 0.338 |
| Waist, mean (SD) |  | 83.8(9.6) | | 88.9(10.6) | <0.001 | 184 (44.4) | 213 (51.4) | 0.214 |
| TC, mean (SD) |  | 4.8(0.9) | | 4.9(1.0) | 0.013 | 4.9(0.9) | 4.9 (0.9) | 0.767 |
| TG, mean (SD) |  | 1.9(1.6) | | 2.4(2.0) | <0.001 | 2.2 (1.7) | 2.3 (2.0) | 0.457 |
| LDL-C, mean (SD) |  | 2.6(0.8) | | 2.6(0.8) | 0.056 | 2.5 (0.8) | 2.6 (0.8) | 0.986 |
| HDL-C, mean (SD) |  | 1.3(0.3) | | 1.3(0.4) | 0.970 | 1.3 (0.4) | 1.3 (0.4) | 0.315 |
| CRP, mean (SD) |  | 5.4(1.4) | | 6.1(2.3) | <0.001 | 5.7 (1.7) | 5.9 (2.2) | 0.062 |

CMM = cardiometabolic multimorbidity, SD= standard deviation, TC = plasma total cholesterol, TG = triglyceride, LDL-C = low density lipoprotein cholesterol, HDL-C = high density lipoprotein cholesterol, CRP = C-reactive protein.

**Table S2**. Associations between plasma homocysteine and specific cardiometabolic diseases^*^.

| **CMD** |  | **Odd Ratio** |  | **95%CI** |  | ***p-value*** |
| --- | --- | --- | --- | --- | --- | --- |
| **Diabetes** |  |  |  |  |  |  |
| Q1 |  | 1.00 |  |  |  |  |
| Q2 |  | 1.03 |  | 0.58-1.85 |  | 0.919 |
| Q3 |  | 0.91 |  | 0.51-1.62 |  | 0.748 |
| Q4 |  | 1.97 |  | 1.15-3.56 |  | 0.013 |
| Trend for per unit |  | 1.08 |  | 1.03-1.13 |  | 0.001 |
| **Hypertension** |  |  |  |  |  |  |
| Q1 |  | 1.00 |  |  |  |  |
| Q2 |  | 2.34 |  | 1.17-4.01 |  | 0.017 |
| Q3 |  | 4.39 |  | 2.17-8.90 |  | <0.001 |
| Q4 |  | 11.35 |  | 5.58-23.09 |  | <0.001 |
| Trend for per unit |  | 1.26 |  | 1.19-1.34 |  | <0.001 |
| **Stroke** |  |  |  |  |  |  |
| Q1 |  | 1.00 |  |  |  |  |
| Q2 |  | 0.56 |  | 0.29-2.65 |  | 0.296 |
| Q3 |  | 0.90 |  | 0.31-2.67 |  | 0.853 |
| Q4 |  | 0.65 |  | 0.29-1.47 |  | 0.297 |
| Trend for per unit |  | 1.00 |  | 0.92-1.05 |  | 0.691 |
| **CHD** |  |  |  |  |  |  |
| Q1 |  | 1.00 |  |  |  |  |
| Q2 |  | 0.90 |  | 0.46-1.76 |  | 0.763 |
| Q3 |  | 1.24 |  | 0.62-2.47 |  | 0.538 |
| Q4 |  | 1.98 |  | 1.05-3.74 |  | 0.034 |
| Trend for per unit |  | 1.06 |  | 1.01-1.12 |  | 0.028 |

CMD = cardiometabolic disease; CHD = coronary heart disease. Using the quartile method to divide HCY into four levels(Q1~Q4).

^*^ All model were adjusted for age, sex, educational attainment, family income, marital status, occupational status, smoking, heavy alcohol consumption, unhealthy diet, inactive exercise, sedentary behavior, fasting blood-glucose, total cholesterol, triglyceride, low density lipoprotein cholesterol, high density lipoprotein cholesterol, and C-reactive protein.

**Table S3**. Correlations between Hcy and potential mediated indicators^*^.

| **Mediated indicators** |  | **Coefficient, β** |  | **95%CI** |  | **P-value** |
| --- | --- | --- | --- | --- | --- | --- |
| CRP |  | 0.0078 |  | 0.0013-0.0142 |  | 0.018 |
| TG |  | 0.0263 |  | 0.0198-0.0328 |  | <0.001 |
| TC |  | 0.0130 |  | 0.0092-0.0168 |  | <0.001 |
| LDL-C |  | 0.0012 |  | -0.0021-0.0046 |  | 0.476 |
| HDL-C |  | -0.0001 |  | -0.0015-0.0012 |  | 0.846 |
| BMI |  | 0.0091 |  | -0.0043-0.0226 |  | 0.183 |
| Waist |  | 0.0492 |  | 0.0108-0.0875 |  | 0.012 |

^*^ Linear models were used and all models were adjusted for age, sex, educational attainment, family income, marital status, occupational status, smoking, heavy alcohol consumption, unhealthy diet, inactive exercise, sedentary behavior.

**Table S4**. Associations between potential mediated indicators and cardiometabolic multimorbidity ^*^.

| **Mediated indicators** |  | **OR** |  | **95%CI** |  | **P-value** |
| --- | --- | --- | --- | --- | --- | --- |
| CRP |  | 1.22 |  | 1.15-1.30 |  | <0.001 |
| TG |  | 1.19 |  | 1.12-1.26 |  | <0.001 |
| TC |  | 1.24 |  | 1.11-1.39 |  | <0.001 |
| LDL-C |  | 0.96 |  | 0.84-1.10 |  | 0.551 |
| HDL-C |  | 0.79 |  | 0.57-1.08 |  | 0.141 |
| BMI |  | 1.14 |  | 1.11-1.18 |  | <0.001 |
| Waist |  | 1.06 |  | 1.05-1.07 |  | <0.001 |

^*^ Logistic models were used and all models were adjusted for age, sex, educational attainment, family income, marital status, occupational status, smoking, heavy alcohol consumption, unhealthy diet, inactive exercise, sedentary behavior.


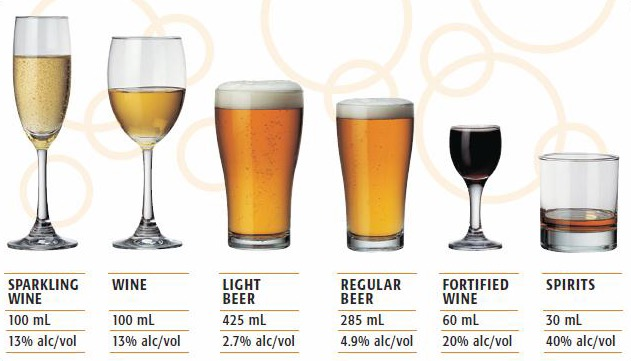


**Fig S1**. **Photos to measure the drinks of different kinds of alcohol drinking.**


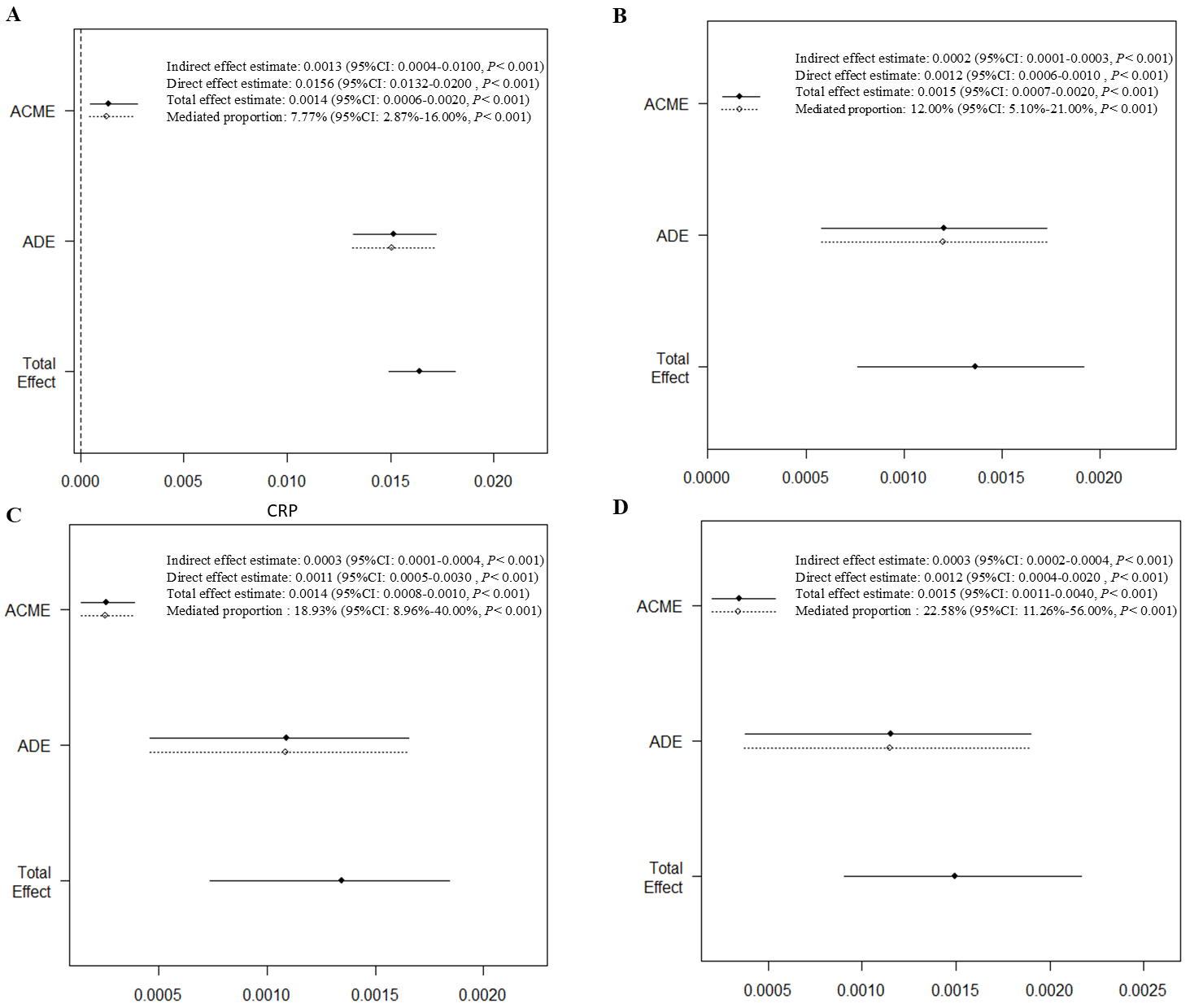


**Fig S2. Mediation analysis of Hcy with CMM.** All models were adjusted for age, sex, educational attainment, family income, marital status, occupational status, smoking, heavy alcohol consumption, unhealthy diet, inactive exercise, sedentary behavior. A, mediation effect by C-reactive protein; B, mediation effect by total cholesterol; C, mediation effect by triglyceride; D, mediation effect by waist.


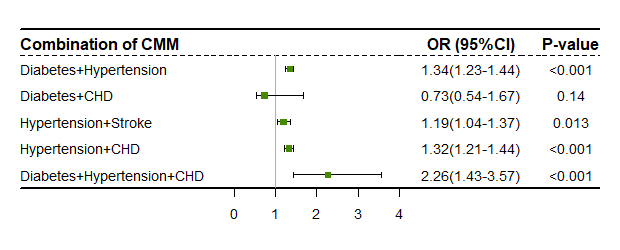


**Fig S3. Associations of Hcy with combination of CMM**. All models were adjusted for age, sex, educational attainment, family income, marital status, occupational status, smoking, heavy alcohol consumption, unhealthy diet, inactive exercise, sedentary behavior.
